# Brain Functional Differences in Drug-Naive Major Depression with Anxiety Patients of Different Traditional Chinese Medicine Syndrome Patterns: A Resting-State fMRI Study

**DOI:** 10.1155/2020/7504917

**Published:** 2020-02-18

**Authors:** Yi Du, Jingjie Zhao, Yongzhi Wang, Yu Han, Ligang Deng, Hongxiao Jia, Yuan Zhou, Joyce Su, Li Li

**Affiliations:** ^1^Department of Traditional Chinese Medicine, Beijing Friendship Hospital, Capital Medical University, Beijing, China; ^2^Your Health & Wellness International Medical Clinic, Beijing, China; ^3^Department of Radiology, Beijing Friendship Hospital, Capital Medical University, Beijing, China; ^4^Department of Psychiatry, Beijing Anding Hospital, Capital Medical University, Beijing, China; ^5^Key Laboratory of Behavioral Science, Institute of Psychology, Chinese Academy of Sciences, Beijing, China; ^6^Department of Psychology, University of Chinese Academy of Sciences, Beijing, China; ^7^Department of Biochemistry, Case Western Reserve University, Cleveland, OH, USA

## Abstract

Major depressive disorder (MDD), especially combined with anxiety, has a high incidence and low detection rate in China. Literature has shown that patients under major depression with anxiety (MDA) are more likely to nominate a somatic, rather than psychological, symptom as their presenting complaint. In the theory of Traditional Chinese Medicine (TCM), clinical symptoms of MDD patients are mainly categorized into two different syndrome patterns: Deficiency and Excess. We intend to use resting-state functional magnetic resonance imaging (rs-fMRI) to investigate their brain functional differences and hopefully to find their brain function mechanism. For our research, 42 drug-naive MDA patients were divided into two groups (21 for Deficiency and 21 for Excess), with an additional 19 unaffected participants in the normal control (NC) group. We took Hamilton Depression Rating Scale (HAMD), Hamilton Anxiety Scale (HAMA), and brain fMRI scan for each group and analyzed the data. We first used Degree Centrality (DC) to map the functional differences in brain regions, utilized these regions as seed points, and used a seed-based functional connectivity (FC) analysis to identify the specific functional connection between groups. The Deficiency group was found to have higher HAMD scores, HAMA scores, and HAMD somatic factor than the Excess group. In the DC analysis, significant decreases were found in the right precuneus of both the Deficiency and Excess groups compared to the NC group. In the FC analysis, the right precuneus showed significant decreased network connectivity with the bilateral cuneus, as well as the right lingual gyrus in the Deficiency group when compared to the NC group and the Excess group. Through our research, it was found that precuneus dysfunction may have a relationship with MDA and Deficiency patients have more severe physical and emotional symptoms, and we realized that a larger sample size and multiple brain mode observations were needed in further research.

## 1. Introduction

Major depressive disorder (MDD) is a mental disorder characterized by low emotion, slow thinking, and reduced speech action. It is a leading cause of global burden (10.3% of the years of life lived with a physical or mental disability), and the prevalence of MDD is 4.45% in the United States and 3.02% in China [1]. MDD patients in China, especially women [2], have been found to complain of somatic symptoms rather than mental symptoms [3]. 60% of Malaysian Chinese depressive patients complained of somatic symptoms, while only 13% of Australian depressive patients did so [4]. This phenomenon may be related to the traditional Chinese viewpoint towards depression, resulting in greater difficulty in identifying and treating MDD patients. As the prevalence rate, recurrence rate, and disability rate of MDD were high [5], major depression with anxiety (MDA) has been confirmed to have more severe physical symptoms and more suicidal thoughts and behavior and are more difficult to be treated [6].

Chinese MDD patients always complain about somatic symptoms, such as stomach pain, arthralgia, or headache [1, 7], and these multiple physical symptoms usually coexist. Traditional Chinese Medicine (TCM) is used to summarize the complex and diverse clinical somatic symptoms by different syndrome patterns and give appropriate treatment. Referring to the internal medicine [8] and clinical study of TCM [9], we chose to research the most important and representative patterns of TCM: Deficiency and Excess. Most somatic symptoms of MDA patients can be summarized by these two patterns and can be easily distinguished (Table 1).

It is difficult to distinguish depression and anxiety and their severity in outpatient quickly, especially in the case of complicated somatization symptoms. Severe MDA patients may have severe impairments, decreased working abilities, and increased risk of incidence rate of comorbid illnesses and may need increased medical treatment [10]. The severity of depression is often determined by psychological scales such as the Hamilton Depression Rating scale (HAMD) [11], 9-item Patient Health Questionnaire (PHQ-9) [12], and Hamilton Anxiety Scale (HAMA) [13]. However, research has showed that such a scale is only effective for short-time illnesses and cannot reflect depression as a chronic illness [14]. Quickly and accurately identifying the severity of depression as a chronic illness is essential for follow-up precise treatment. Our research focused on the brain function mechanism differences between TCM patterns in MDA patients and provided theoretical support for syndrome treatment of TCM.

Resting-state functional magnetic resonance imaging (rs-fMRI) is a noninvasive way to observe brain function and is thus widely used by researchers of brain and mood disorders, including depression [15]. The study of MDD patient brain function showed that there are regions, connections, and network differences from those without MDD. The brain regions which are related to depression are located in the cingulate gyrus [16, 17], prefrontal cortex, cuneus [18, 19], insula [20], and gyrus lingualis [21], as well as the default-mode network (DMN) in brain-resting networks [22]. The amygdala [23] and hippocampus [24] were also areas of interest. These regions and networks are related to cognition, memory, and emotion and confirmed the brain function mechanism of depressive patients [25]. Compared to MDD, there were also several functional abnormal regions in MDA patients such as ventral anterior cingulate and amygdala [26], middle temporal gyrus and cuneus [27], and even functional connection abnormal in DMN [28]. Although many functional abnormal brain regions were found in both MDD and MDA, they were relatively concentrated and mostly related to emotional and cognitive function.

Therefore, our hypothesis is that there are even brain functional differences between Deficiency and Excess patterns of MDA patients in these brain regions, and these differences result in different somatic symptoms and TCM patterns. So we intent to use rs-fMRI to research the brain function differences between Deficiency and Excess MDA patients to describe brain function mechanisms of these two TCM patterns and to provide clinical materials to support follow-up TCM syndrome differentiation and treatment of MDA.

## 2. Materials and Methods

### 2.1. Subjects

The MDA patients were screened from the clinics of Beijing Friendship Hospital and Beijing Anding Hospital. Normal controls were recruited from the local community through advertisements. We screened 42 patients that met the two TCM patterns from 63 first-episode treatment-naive MDA patients (21 Deficiency patients and 21 Excess patients, defined according to TCM syndrome patterns) and recruited 19 matched normal controls (NC). All patient-involved activities were preapproved by the Medical Research Ethics Committee of Beijing Friendship Hospital, Capital Medical University. Written informed consent was obtained from each participant.

For all participants, the inclusion criteria were as follows: (1) between the age of 18 and 65 years; (2) right-handed; and (3) have a Mini-Mental State Examination (MMSE) score >24 [29, 30]. Participants were excluded if they had (1) primary neurological illness, including dementia or stroke; (2) any brain white matter changes in T2-weighted magnetic resonance images, including infarction or other vascular lesions and gray matter atrophy; (3) history of any other major psychiatric diseases, such as bipolar disorder, schizophrenia, personality disorder, intellectual disability, and claustrophobia; (4) presence of a medical illness that could impair cognitive functions, such as diabetes; (5) alcohol/drug abuse or dependence; and (6) metallic foreign bodies such as pacemakers, metallic dentures, or amalgam fillings. In addition to complying with the aforementioned general criteria, MDA subjects were subjected to the following additional inclusion criteria: (1) diagnoses by way of structured clinical interviews by two well-trained senior psychiatrists in accordance with the DSM-IV criteria for depressive disorder with anxious distress [31]; (2) 24-item HAMD scores ≥18; (3) Hamilton Anxiety Scale (HAMA) score ≥14; (4) conform to the diagnostic criteria of Deficiency and Excess of TCM syndrome differentiation standard [8]; and (5) medication-free for at least 2 weeks. The additional criteria for normal controls were as follows: (1) HAMD score <8 and (2) HAMA score <7.

### 2.2. MRI Data Acquisition

Images were acquired with a 3.0 Tesla GE MRI scanner (SIGNA EXCITE) from the Radiology Department of Beijing Friendship Hospital. The subjects were told to keep their eyes closed and minds relaxed during the scanning and to not fall asleep.

Whole-brain functional scans were collected in 34 axial slices using an echo-planar matrix = 64 × 64, field of view = 220 × 220 mm^2^, slice thickness = 4 mm, and slice gap = 0.5 mm. Each functional run contained 240 volumes.

### 2.3. Imaging Data Preprocessing

Unless otherwise stated, all preprocessing was performed using the Data Processing Assistant for Resting-State fMRI [32], which is based on the Statistical Parametric Mapping (SPM12) [33] program and the Resting-State fMRI Data Analysis Toolkit [34]. Prior to preprocessing, the first 5 volumes were discarded to allow for signal stabilization. The remaining volumes acquired from each subject were corrected for the differences in slice acquisition times. The resultant images were then realigned to correct for small movements that occurred between scans. The resulting maps were then registered into the Montreal Neurological Institute Atlas space with an EPI template, resampling to 3 mm isotropic voxels. A 6 mm full-width half-maximum Gaussian kernel was used for spatially smoothing. Then, several sources of spurious or regionally nonspecific variance were removed from the data by regression of nuisance variables including (1) 24 parameters (including 6 head motion parameters, 6 head motion parameters one time point before, and the 12 corresponding squared items) obtained by rigid body head motion correction, (2) the signal averaged over the whole-brain (global signal), (3) the signal averaged over the lateral ventricles, (4) the signal averaged over a region centered in the deep cerebral white matter, and (5) linear and quadratic trends [35]. Temporal filtering (0.01–0.1 Hz) of the time series was then performed. The volume-based mean framewise displacement (FD), comparing head position variations between the current and previous volumes, was used to quantify head motion across the volumes for each participant [36].

#### 2.3.1. Degree Centrality

Individual degree centrality (DC) maps were generated in a voxel-wise way within a study mask, which is a predefined gray matter mask including tissue with gray matter probabilities greater than 20% as previously described [37]. First, the preprocessed functional runs were subjected to voxel-based whole-brain correlation analysis. The time course of each voxel from each participant that was within the gray matter mask was correlated with the time course of every other voxel, which resulted in a correlation matrix. An undirected adjacency matrix was then obtained by thresholding each correlation at *r* > 0.25 [37–39]. Then, the DC was computed as the number of significant correlations (binarized) or as the sum of the weights of the significant connections (weighted) for each voxel. Finally, the individual-level voxel-wise DC was converted into a *z*-score map by subtracting the mean DC across the entire brain and dividing by the standard deviation of the whole-brain DC [35, 37].

#### 2.3.2. Seed-Based Functional Connectivity

After identifying the region(s) whose DC showed significant across-group differences, we used a seed-based functional connectivity (FC) analysis to identify the specific functional connectivity contributing to the across-group differences. Specifically, the mean time series of each seed region was acquired by averaging the time series of all of the voxels within that region. And then the correlation coefficients between the averaged time course of the seed region with all other voxels in the brain were computed. Finally, the correlation coefficients were converted into *z*-values using Fisher's r-to-z transformation to improve their normality.

### 2.4. Statistics

One-way ANOVA analysis was conducted to test whether there were any differences in DC or FC while taking the head motion measured by the mean FD as no interests of covariates.

Standard error method was used for data analysis, and significant group differences were obtained with a cluster-wise FWE corrected *P* value of 0.05 for multiple comparisons (individual voxel threshold, *P* < 0.001). If the main effect was statistically significant, simple effect analyses were performed for the averaged effect size extracted from the clusters with significant effects using SPSS v19.0.

## 3. Results

### 3.1. Demographic and Clinical Data

There were no significant differences in age, dender, educational years, and head motion measured by the mean FD and the number of scrubbing of bad time points. However, we do see significant difference in the HAMD score, HAMA score, and HAMD somatic factor between Deficiency and Excess groups (Table 2).

### 3.2. Degree Centrality

**Figure 1:**
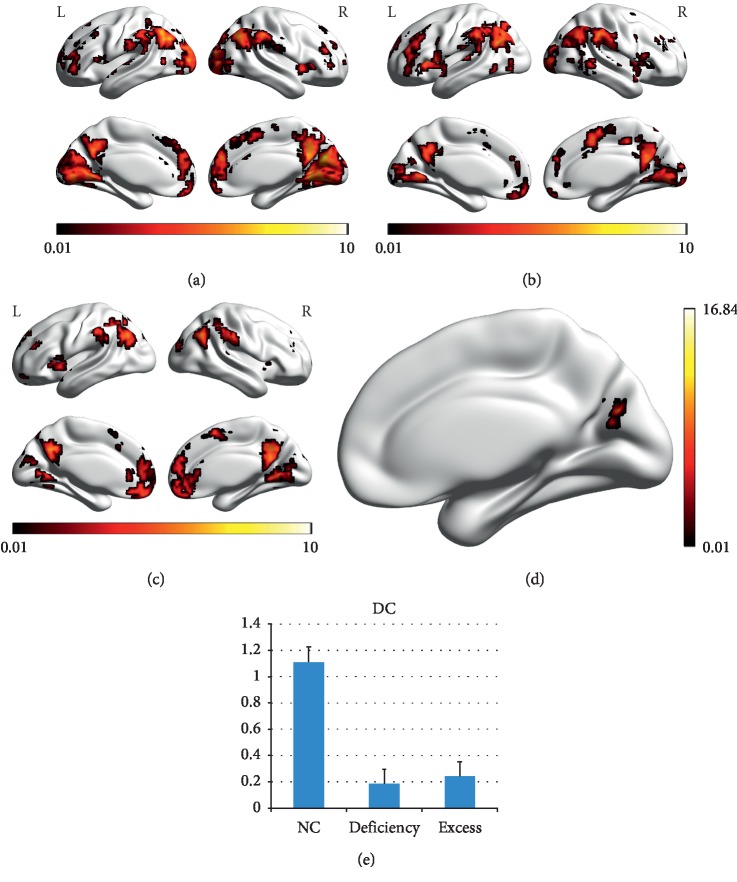
Degree centrality (DC) in each group and differences across groups. (a) DC in the NC group, (b) DC in the Deficiency group, (c) DC in the Excess group, (d) ANOVA results, and (e) Post hoc comparisons. The dark and light colors indicate the *P* value. The deeper the color, the smaller the *P* value.

In the NC group, the spatial distribution of the weighted DC was highly localized in the posterior cingulate/ventral precuneus, occipital lobe, middle cingulate cortex (MCC), anterior cingulate cortex/medial prefrontal cortices, lateral prefrontal cortex, inferior parietal regions, insula (Figure 1(a)). Both in the Deficiency and Excess groups, the spatial distribution of the weighted DC was also localized in the abovementioned regions, but the clusters were smaller (Figures 1(b) and 1(c)). Compared to the NC group, significant decreases in the weighted DC were found in the right precuneus in both of the Deficiency group and Excess group (Figures 1(d) and 1(e), Table 3).

### 3.3. Seed-Based Functional Connectivity

**Figure 2:**
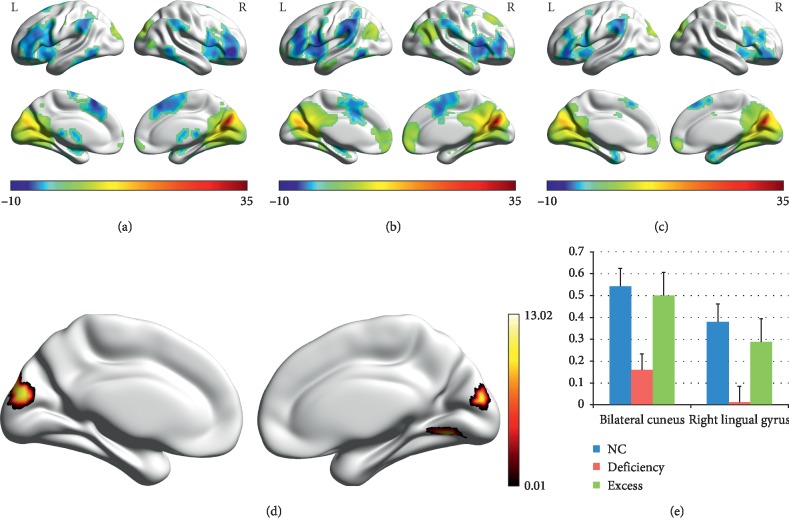
Functional connectivity (FC) of the right precuneus in each group and differences across groups. (a) DC in the NC group, (b) DC in the Deficiency group, (c) DC in the Excess group, (d) ANOVA results, and (e) post-hoc comparisons. The warm and cold colors in (a–c) indicate the brain regions with significantly increased and decreased FC. The dark and light colors in (d) indicate the *P* value; the deeper the color, the smaller the *P* value.

We used the right precuneus which showed significance between group differences as the seed regions for the mapping of the functional connectivity network. In general, the right precuneus showed significant positive connectivity with the regions in a default-mode network (DMN), such as the posterior cingulate cortex and medial prefrontal cortex, and showed significant negative connectivity with the regions in an executive control network (ECN), such as the bilateral prefrontal cortex and parietal cortex, and the regions in a salience network (SAN), such as the bilateral anterior insula and cingulate cortex, in each group (Figures 2(a)–2(c)). ANOVA found significant differences in the connectivity between the right precuneus and the bilateral cuneus as well as the right lingual gyrus (Figure 2(d)). Post-hoc comparisons revealed that the connectivity was decreased in the Deficiency group compared to the NC group and the Excess group, but there were no differences between the Deficiency group and the NC group (Figure 2(e) and Table 4).

## 4. Discussion

TCM has a history of more than 2,000 years. It is a medical theory system that has been gradually formed and developed through long-term medical practice under the guidance of simple materialism and dialectical thinking. Deficiency and Excess are two most important TCM types with outstanding symptoms in MDD patients [40, 41] and usually to be studied as typical syndrome patterns of MDD [42, 43], so we choose them for observation.

According to the scale analysis, we found that the HAMD scores, HAMA scores, and HAMD somatic factor of the Deficiency group were much higher than those of the Excess group. This result had been reported by previous researches [44, 45]. They suggest that the Deficiency patients had longer disease course and more serious somatic anxiety. Therefore, these two different TCM syndrome patterns can reflect the severity of MDA to a certain extent.

We used the weighted DC to analyze the rs-fMRI data, which demonstrated that all the three groups' spatial distributions of the weighted DC were highly localized in the DMN and dorsal attention network (DAN). These brain networks play a central role in resting-state research [46, 47]. Then when we used the right precuneus as a seed to calculate functional connectivity, it showed significantly positive connectivity with the regions in DMN and negative connectivity with the regions in ECN and SAN in general. These distributions are similar to those reported by previous studies [48–50]. It may be indicated that the function of self-consciousness [51], spontaneous thinking [52], and self-related processes [53] were deteriorated in MDA patients.

At the same time, we found that the precuneus was a very important region for MDA patients. Compared with the NC participants, both Deficiency and Excess patients had decreased brain function in the right precuneus. Previous studies have also shown that depressive patients have abnormal brain function in the precuneus [54, 55]. This region is very important for self-reflection processes and potentially plays a role in mental imagery and episodic/autobiographical memory retrieval [56–58]. The precuneus also assists other brain regions in performing functions such as information processing, especially in regards to emotion regulation [59, 60]. Furthermore, the precuneus is a major hub of brain organization and a central node of DMN [61, 62]. It is involved in a variety of information processing states [63, 64]. There were additional reports that the depressive patients' precuneus made functional connectivity separation of the brain network and that dissociated large-scale networks may have contributed to the clinical expression of depression [65]. Although we found that both Deficiency and Excess groups' patients have decreased function in the right precuneus in comparison with the NC group, there were no significant differences between the two TCM syndrome pattern groups. It indicates that the dysfunction of the right precuneus may be the manifestation of brain function abnormality in MDA patients.

From our study, compared with NC and Excess groups, the Deficiency group showed negative connectivity with the bilateral cuneus as well as the right lingual gyrus. There are also abnormal functions of these two brain regions to be found in panic disorder patients [66]. While the cuneus has a function of integrating the somato-sensory information with other sensory stimuli and cognitive processes such as attention, learning, and memory [67], the lingual gyrus is a brain region responsible for supporting visual memory [68]. Furthermore, the precuneus was also activated in many version tasks [69, 70]. Research had also shown that depression and anxiety are primarily correlated with functional deficits in the precuneus-related network [71].

However, we also notice that we need larger samples and even multiple brain mode observations to confirm if the two TCM syndrome patterns have more significant brain regions and network functional differences in precuneus or other brain regions in future research.

## 5. Conclusions

In summary, there are differences in brain function between the two different TCM syndrome patterns of MDA patients. Based on our research, it was found that precuneus dysfunction may have a relationship with MDA and brain functional connectivity differences, and we could find out that the Deficiency patients have more severe physical and emotional symptoms in MDA patients. At the same time, we realized that a larger sample size and multiple brain mode observations were needed in further research.

## Figures and Tables

**Table 1:** Syndrome characteristics of the two TCM syndromes.

TCM pattern	Syndrome characteristic	Tongue image and pulse
Deficiency	Thoughtful, suspicious, dizzy, timid, heart palpitations, insomnia, forgetfulness, loss of appetite, pale complexion	Pale tongue, thin white fur, weak pulse

Excess	Depression, impatience, chest swell, flank rib pain, suffocating, inappetence, stool irregularities	Thin and greasy fur, string pulse

**Table 2:** Demographic and clinical characteristics of the participants.

	NC (*n* = 19)	Deficiency (*n* = 21)	Excess (*n* = 21)	*P* value
Age	46.79 ± 13.97	39.62 ± 12.39	46.38 ± 13.28	0.151
Gender (M/F)	4/15	6/15	4/17	0.585
Education (years)	12.52 ± 3.45	11.90 ± 2.93	11.28 ± 2.77	0.444
HAMD score	—	29.24 ± 6.65	24.76 ± 6.54	0.034
HAMA score	—	26.48 ± 5.24	19.81 ± 5.20	0.000
HAMD somatic factor	—	9.14 ± 1.49	5.33 ± 1.31	0.000
Head motion: mean FD	0.15 ± 0.04	0.13 ± 0.07	0.14 ± 0.04	0.276

HAMD, Hamilton Depression Rating Scale; HAMA, Hamilton Anxiety Rating Scale; NC, normal controls.

**Table 3:** DC differences in the three groups.

Cluster location	NC	Deficiency	Excess	*P* value		
				NC vs. Deficiency	NC vs. Excess	Deficiency vs. Excess
Right precuneus	1.11 ± 0.51	0.18 ± 0.52	0.24 ± 0.51	<0.00	< 0.001	0.712

DC, degree centrality; NC, normal controls.

**Table 4:** FC differences in three groups.

Cluster location	NC	Deficiency	Excess	*P* value		
				NC vs. Deficiency	NC vs. Excess	Deficiency vs. Excess
Bilateral cuneus	0.54 ± 0.36	0.16 ± 0.26	0.50 ± 0.27	<0.001	0.647	<0.001
Right lingual gyrus	0.38 ± 0.32	0.01 ± 0.19	0.29 ± 0.27	<0.001	0.272	<0.001

FC, functional connectivity; NC, normal controls.

## Data Availability

All data generated or analyzed during this study are included within the article.

## Abbreviation

MDD: Major depressive disorder
MDA: Major depression with anxiety
TCM: Traditional Chinese Medicine
rs-fMRI: Resting-state functional magnetic resonance imaging
DC: Degree Centrality
FC: Functional connectivity
HAMD: Hamilton Depression Rating Scale
HAMA: Hamilton Anxiety Scale
DMN: Default-mode network
ECN: Executive control network
SAN: Salience network.
